# Opioid Use Among Rural Medicare Beneficiaries

**DOI:** 10.1093/geroni/igab046.1294

**Published:** 2021-12-17

**Authors:** Yvonne Catharina Jonk, Heidi O'Connor, Karen Pearson, Zachariah Croll, John Gale

**Affiliations:** 1 University of Southern Maine, Muskie School, Portland, Maine, United States; 2 University of Southern Maine, Portland, Maine, United States; 3 University Of Southern Maine, Portland, Maine, United States

## Abstract

This study examines differences in opioid prescribing rates among a nationally representative sample of Medicare beneficiaries across rural and urban areas, as well as among beneficiaries with chronic overlapping pain conditions (COPCs). We assess whether prescribing patterns exceed the Centers for Disease Control and Prevention guidelines for dose and duration, and identify socioeconomic and health risk factors associated with opioid prescribing using logistic regression analyses. Data were from the 2010-2017 Medicare Current Beneficiary Survey files. Rural-Urban Commuting Area codes were used to identify patients’ residential location. The Area Health Resource Files were used to identify market characteristics such as primary care and mental health shortage areas. With the exception of 2010, over years 2011-2017, higher percentages of community-dwelling rural beneficiaries received opioid prescriptions (21.8-25.4%) compared to their urban counterparts (19.1-23.7%). During the same time period, facility-dwelling rural beneficiaries were more likely to receive opioid prescriptions (39.8-47.2%) compared to their urban counterparts (28.8-35.0%). Higher percentages (18.8%) of the community dwelling population in rural had COPCs compared to urban (15.2%), and a higher percentage of rural beneficiaries with COPCs (31.4%) received an opioid prescription than their urban counterparts (22.2%). Previous research points to other factors contributing to a lack of alternatives to opioids for pain management in rural areas, including greater reliance on primary care providers, lack of access to chronic pain specialists and alternative therapies, and travel barriers. Improving the capacity of rural primary care to deal with COPCs and expanding access to specialists via telehealth warrants further attention from policymakers.

